# Incidental transmural granulomatous enteritis caused by *Molineus torulosus* in a capuchin monkey (*Sapajus nigritus*) under human care: pathological and morphometric findings

**DOI:** 10.1007/s11259-026-11501-z

**Published:** 2026-09-07

**Authors:** Fernanda de Freitas Alves Vieira, Larissa Megumi Nogueira Sato, Mylena Oliveira Miranda, Sheila Canevese Rahal, José Gabriel Gonçalves Lins, Didier Quevedo Cagnini

**Affiliations:** School of Veterinary Medicine and Animal Science, São Paulo State University - UNESP, Botucatu, São Paulo Brazil

**Keywords:** Nematoda, Granulomatous inflammation, Wild animals, Gastrointestinal pathology

## Abstract

Primates of the genus *Sapajus* are widely distributed throughout Brazil, with several species currently listed as endangered by the IUCN. Identifying diseases that affect these populations is critical, particularly parasitic infections, which can debilitate hosts and predispose them to secondary pathogens. *Molineus torulosus* is a trichostrongylid nematode known to cause significant gastrointestinal injury in neotropical primates. This report describes the pathological and morphometric findings of an incidental case of *M. torulosus* parasitism in a captive female capuchin monkey (*Sapajus nigritus*) referred to the postmortem examination. The animal was admitted to the Center for Medicine and Research of Wild Animals, presenting perforating skin lesions and progressive spastic paralysis, under clinical suspicion of tetanus. Following the animal’s death three days post-admission, gross examination revealed poor body condition and ulcerative skin lesions. The primary gross finding consisted of multiple whitish, firm, transmural nodules in the duodenum and jejunum. Histopathological evaluation of these nodules revealed multifocal, moderate, lymphohistiocytic and granulomatous enteritis associated with intralesional nematodes and eggs; meanwhile, the central nervous system showed no lesions, supporting the clinical suspicion of tetanus. Parasitological examination confirmed the agent as *M. torulosus*. This case highlights the severe pathological impact of this parasite and emphasizes the importance of diagnostic surveillance and health monitoring in wild animals under human care to expand the limited literature on parasitic diseases in Neotropical primates.

## Background

The genus *Sapajus* comprises New World neotropical primates that are widely distributed throughout Brazil (Lynch et al. 2012). However, these primates face different threats, including habitat degradation, fragmentation, and loss, as well as hunting and illegal trade (Bueno et al. 2017). Habitat fragmentation, in particular, increases disease susceptibility in primate populations, while the reduction of population sizes in isolated forest patches further increases the risk of epidemics (Smith et al. 2009). As several species within this genus are currently listed as threatened or endangered by the International Union for Conservation of Nature (IUCN), identifying the diseases affecting these populations is crucial for effective conservation and management strategies.

Parasitic infections represent a key, yet often underrecognized, component of wildlife health (Jenkins et al. 2015). Among the pathogens affecting these primates, gastrointestinal nematodes, such as *Molineus torulosus*, are of particular concern due to their potential to cause severe enteritis and impact the overall fitness of captive and free-ranging populations, especially under environmentally stressed conditions (Miguel et al. 2013; Bacalhao et al. 2016; Ehlers et al. 2022). Therefore, this report aims to describe the pathological and parasitological findings by *M. torulosus* infection in a capuchin monkey under human care (*Sapajus nigritus*), contributing to the understanding of its clinical and pathological significance.

## Case presentation

An intact adult female capuchin monkey (*Sapajus nigritus*), weighing 2.1 kg, from a zoo located in São Paulo State, was admitted to a Center for Wildlife Medicine and Research (CEMPAS). The clinical history included progressive spastic paralysis starting in the thoracic limbs, weight loss, and cutaneous perforating lesions. The animal died three days after admission, and a post-mortem examination was performed under clinical suspicion of tetanus. This suspicion was based on the clinical examination and signs observed in the animal and supported by the history of a previous tetanus case in an *Alouatta* spp. four years earlier at the same institution.

**Fig. 1:**
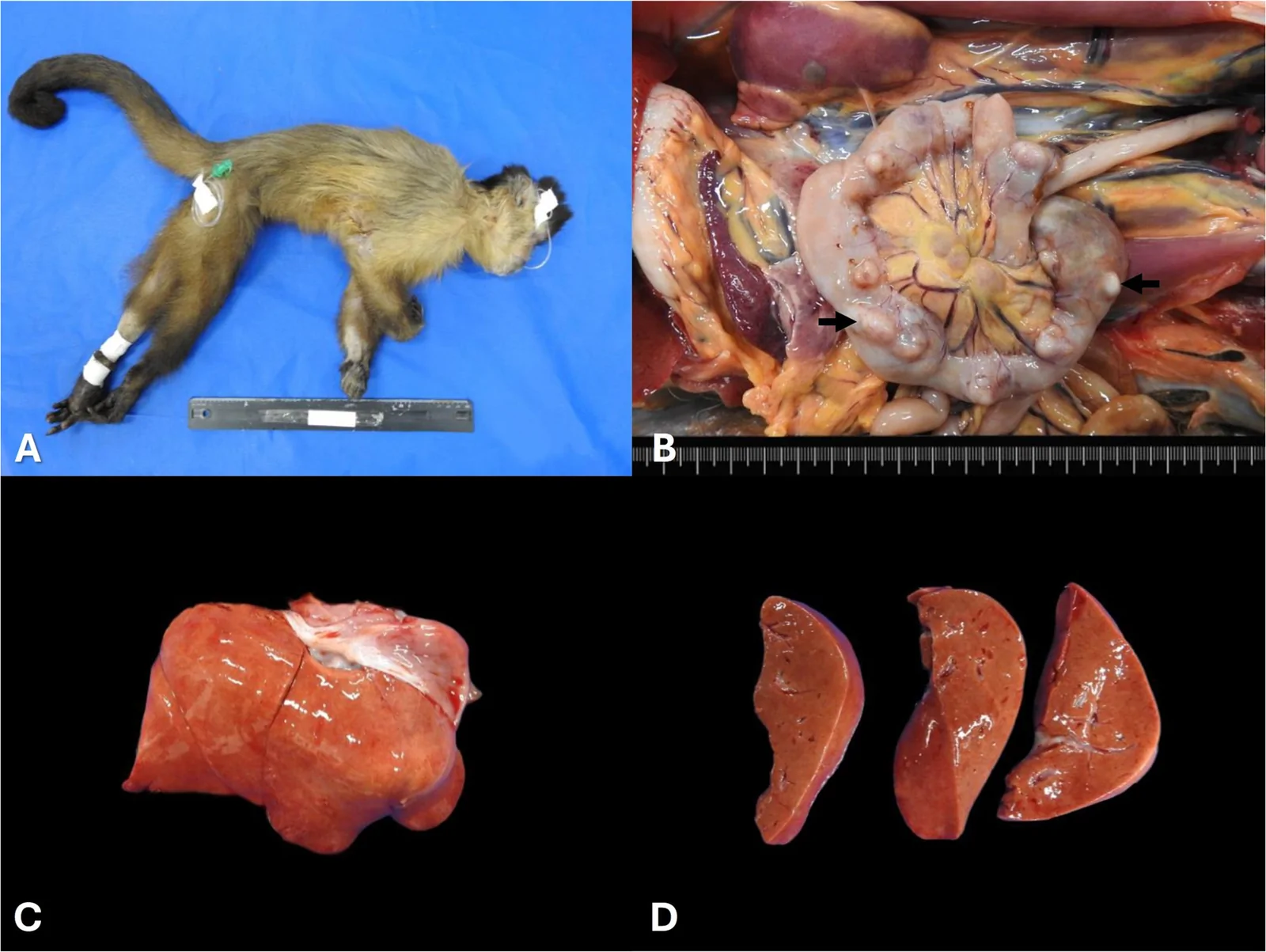
Gross findings of *Molineus torulosus* infection in a capuchin monkey (*Sapajus nigritus*). (**A**) Lateral view of the animal at external examination showing emaciation. (**B**) Segment of the jejunum exhibiting multiple transmural, firm, and whitish nodules (arrow), ranging from 0.3 to 0.7 cm in diameter. The nodules are well-circumscribed and irregular, visible from the serosal surface. (**C**) Dorsal and (**D**) cut surface of the liver are diffusely light brown and greasy

At post-mortem examination, the capuchin monkey (Fig. 1A) was in poor body condition (emaciated). The most significant lesions were in the small intestine, where segments of the duodenum, jejunum, and ileum exhibited multiple transmural, whitish nodules visible from the serosal surface. These nodules ranged from 0.3 to 0.7 cm in diameter and were well-circumscribed, irregular, and firm (Fig. 1B). Upon sectioning, the nodules were solid and uniformly white. The liver was moderately greasy, and diffusely light brown morphologically compatible fatty liver degeneration (Fig. 1C and D).

After necropsy, tissue samples were collected for routine histopathological evaluation, fixed in 10% buffered formalin, and processed for Hematoxylin and Eosin (H&E) staining. For the parasitological examination, a 10-cm segment of the small intestine exhibiting transmural nodules was selected and processed for helminth recovery using a digestion technique.

Briefly, the intestinal sections were opened, and its content was recovered into a Petri dish. The nodules within the selected intestinal segment were then sectioned, and the tissue was soaked in a 0.9% saline solution (NaCl) at 27 °C for 12 h. The digested material was transferred to a conical sedimentation glass and stored at 4 °C for 24 h. After discarding the supernatant, the sediment was examined under a stereomicroscope. All nematodes recovered from the digested material were counted, and specimens were morphologically identified according to their developmental stage. Recovered nematodes were temporarily mounted on glass slides and cleared in a solution of 70% ethanol and 10% glycerin for morphological identification.

**Fig. 2:**
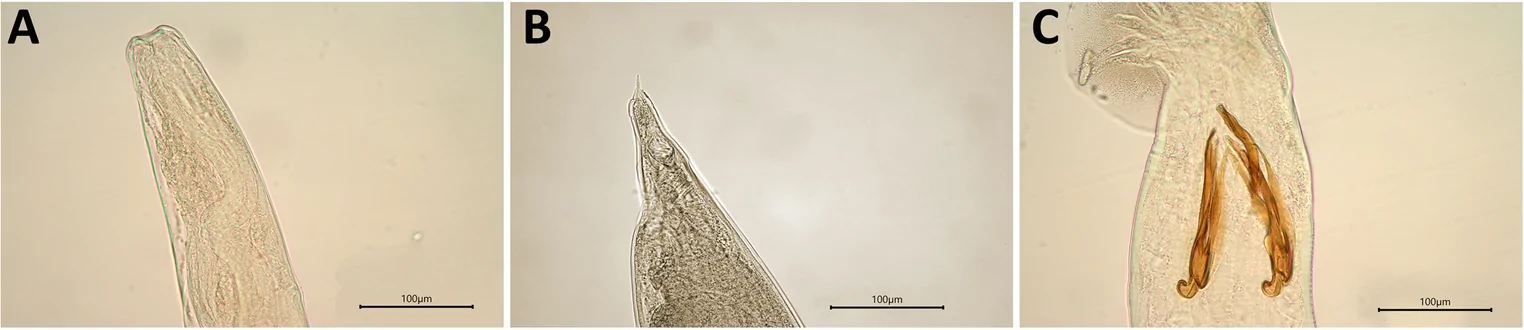
Morphological features of *Molineus torulosus* recovered from a captive capuchin monkey (*Sapajus nigritus*). (**A**) Anterior region showing an elongated, slightly tapered extremity with smooth cuticle, subtle longitudinal striations, and a terminal oral opening. (**B**) Posterior region of an adult female with a long tail ending in a caudal spine. (**C**) Posterior region of an adult male showing a symmetrical caudal bursa (2–1–2), alate spicules, and a keratinized gubernaculum

A total of four nematodes (two adult males and two adult females) were recovered from the intestinal sediment. The parasites were identified as *M. torulosus* (Family: Molineidae), based on established taxonomic keys (Vicente et al. 1997; Durette-Desset 2001). The anterior end is elongated and slightly tapered, with a smooth cuticle and subtle longitudinal striations. The oral opening is terminal, and the esophageal region extends posteriorly (Fig. 2A). Adult females were characterized by the presence of an excretory pore and a long tail terminating in a distinct caudal spine (Fig. 2B). Morulated eggs were observed within the uterine branches. Adult males exhibited a symmetrical caudal bursa with a type 2-1-2 ray pattern. The spicules were long (159,35 μm and 162,34 μm) and alated, with a handle shorter than blade, and were accompanied by a keratinized gubernaculum (101,25 μm). Both spicules and the gubernaculum showed characteristic keratinized (Fig. 2C).

**Fig. 3:**
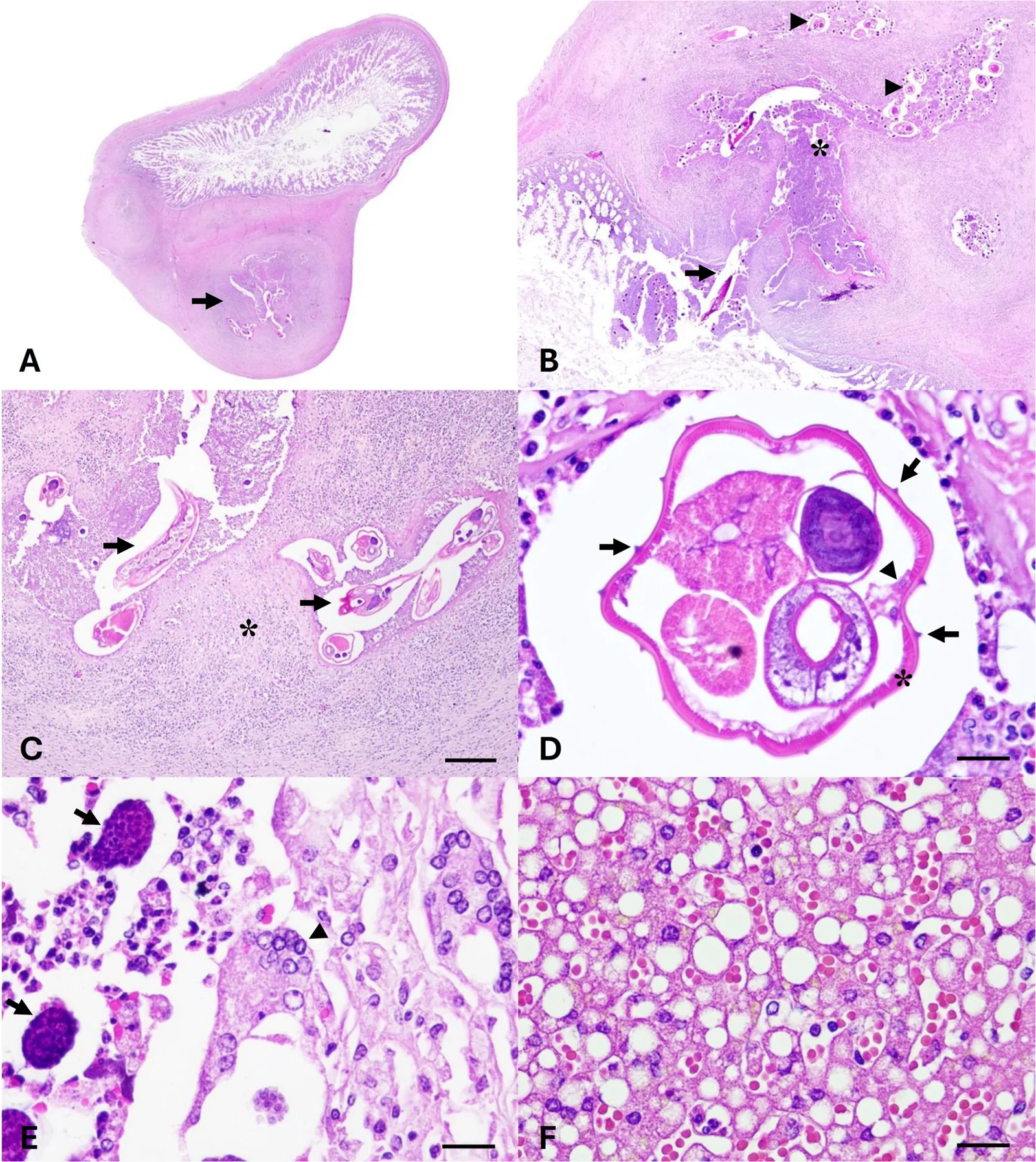
Histopathological findings of *Molineus torulosus* infection in a capuchin monkey (*Sapajus nigritus*). (**A**) Subgross view of the small intestine presenting transmural granulomas with a necrotic center (arrow). H&E stain. (**B**) Fistulous tract (arrow) communicating the granulomatous necrotic core (asterisk) with the intestinal lumen, showing the release of morulated eggs and an adult nematode. Note the presence of nematodes in transverse section (arrowhead) and their eggs. H&E stain, bar = 500 μm. (**C**) Detail of the granulomatous area showing intralesional nematodes (arrow) and eggs within the necrotic core (asterisk). H&E stain, bar = 200 μm. (**D**) Cross-section of an adult nematode within a granuloma, showing a thick cuticle with longitudinal ridges (synlophe) (arrow), lateral cords (arrowhead), platymyarian musculature (asterisk), and internal organs. H&E stain, bar = 20 μm. (**E**) Granulomatous infiltrate composed of eosinophils, macrophages, lymphocytes, and multinucleated giant cells (arrowhead) surrounding two parasitic eggs. H&E stain, bar = 20 μm. (**F**) Moderate to marked hepatocellular steatosis with discrete intracytoplasmic yellow-brown pigment accumulation (cholestasis). H&E stain, bar = 20 μm

Histopathology revealed a multifocal, moderate, transmural granulomatous and ulcerative enteritis with intralesional eggs and adult nematodes morphologically consistent with *Molineus torulosus* (Fig. 3A-E). The lesions were characterized by a necrotic core containing cellular debris, eggs, and adult nematodes, surrounded by an inflammatory infiltrate composed of epithelioid macrophages, multinucleated giant cells, lymphocytes, and plasma cells, all encapsulated by a thin layer of fibrous connective tissue (Fig. 3A-C). The adult nematodes exhibited a 5-µm-thick hyaline eosinophilic cuticle with external longitudinal ridges (synlophe), platymyarian-coelomyarian musculature, a pseudocoelom, and both digestive and reproductive tracts (Fig. 3D). The eggs, measuring 27–36 μm in diameter, featured a thin shell and contained morulae (Fig. 3E). Furthermore, fistulous communication between the granulomatous core and the intestinal lumen was observed, allowing the passage of eggs interspersed with amorphous material and cellular debris (Fig. 3B). The liver presented with diffuse hepatocyte vacuolation characterized by numerous large intracytoplasmic vacuoles with nuclear marginalization (steatosis) and yellow-brown pigment accumulation (cholestasis). These empty spaces were negative for PAS stain (Fig. 3F). The skin revealed a locally extensive area of epidermal ulceration, associated with coagulative necrosis of the underlying dermis and multifocal hemorrhage, and was covered by a thick serocellular (fibrinoleucocytic) crust composed of abundant fibrin, cellular debris, and both intact and degenerated neutrophils. Microscopic evaluation of the brain and spinal cord was unremarkable.

## Discussion and conclusions

The alimentary tract of nonhuman primates, particularly in wildlife, is frequently affected by nematodes across the oral cavity, esophagus, stomach, and intestines (Kouassi et al. 2015; Terio et al. 2018; Vonfeld et al. 2022; Silva et al., 2024), as observed in the present case. Within the small intestine parasites of the order Strongylida are prevalent, with the genera *Trichostrongylus*, *Molineus*, *Strongyloides*, *Ascaris*, and *Ancylostoma* spp. being the most observed (Toft, 1982).

*M. torulosus* has been reported in several neotropical primates, including *Cebus apella*, *Cebus capucinus*, *Cebus libidinosus*, *Saimiri sciureus*, as well as species within the genera *Cebus* and *Sapajus*, indicating a relatively broad host range among New World primates (Corrêa et al. 2016). Although reported in different regions of Brazil, its occurrence is not restricted to this country, being documented in other regions of the South America, such as French Guyana (Durette-Desset 2001). The preferred host-parasite site is the upper small intestine, particularly the duodenum and jejunum, but it may also be found throughout other intestinal segments (Toft 1982; Strait et al. 2012). Infection is typically acquired through ingestion of infective third-stage larvae (L3) in the environment, following a direct life cycle (Durette-Desset 2001). After infection, L3 penetrates the intestinal mucosa and migrates into deeper layers, where they induce the formation of submucosal granulomas (Durette-Desset 2001). Within these structures, larvae develop into adults, copulate, and produce eggs (Durette-Desset 2001). These cysts maintain communication with the intestinal lumen through narrow channels, allowing the release of eggs, which are subsequently excreted in the feces. In the environment, eggs hatch and develop into infective stages, completing the life cycle (Durette-Desset 2001).

Clinical signs are often nonspecific, including diarrhea, progressive weight loss, and marked debilitation (Strait et al. 2012; Miguel et al. 2013; Bacalhao et al. 2016). In this context, the low body weight observed in the present case may be directly associated with *M. torulosus* infection, likely resulting from chronic intestinal damage and impaired nutrient absorption.

Grossly, nematodes that induce lesions similar to those of *Molineus* sp. include *Oesophagostomum* sp. (cecum and colon) and *Nochtia nochtia* (stomach and intestine), especially in Old World monkeys (Kouassi et al. 2015; Terio et al. 2018; Silva et al. 2024). In New World primates, differential diagnoses should include acanthocephalans such as *Prosthenorchis* sp. in the ileum, cecum, and colon, and trematodes like *Schistosoma mansoni* in the colon (Toft 1982; Bogers et al. 2004; Rojas-Sánchez et al. 2023). Furthermore, granulomatous lesions associated with *Mycobacterium tuberculosis* must be considered, as they can affect multiple organs, including the small intestine (Gong et al. 2017).

As identified in the present case, the infection by *M. torulosus* is associated with ulcerative and granulomatous enteritis, characterized by the formation of cyst-like nodules within the intestinal wall (Strait et al. 2012; Bacalhao et al. 2016; Ehlers et al. 2022). In *Cebus* spp., severe cases may result in hemorrhagic or ulcerative enteritis, fibrotic nodules, and, in advanced stages, complications such as peritonitis, septicemia, and death (Miguel et al. 2013). Additionally, erratic migration in the host has been described, with larvae potentially invading vascular structures and reaching organs such as the pancreas, where they may contribute to chronic inflammatory processes (Miguel et al. 2013).

Although the *M. torulosus* infection was considered an incidental finding in this case, granulomatous enteritis associated with a high parasite burden can impair intestinal function and nutrient absorption, potentially leading to weight loss and emaciation (Bacalhao et al. 2016; Silva et al. 2024). Severe malnutrition triggers the mobilization of peripheral fat stores as an energy source, which may exceed the metabolic capacity of the liver and inducing hepatic lipidosis (van Zutphen et al. 2016). A similar process may have contributed to the poor body condition and hepatic lipidosis observed in this case.

Tetanus was considered the probable cause of death based on the clinical presentation of spastic paralysis, skin perforating wounds, and the absence of histopathological lesions in the central nervous system. No additional tests were conducted to confirm this diagnosis.

This report highlights the pathological and morphometric findings of granulomatous enteritis caused by *Molineus torulosus* in a capuchin monkey. The morphological characterization of the parasite in H&E-stained sections and through parasitological morphometry contributes to accurate diagnosis and supports the correct identification of the parasite. Although considered an incidental finding, the extensive intestinal lesions observed suggest that *M. torulosus* infection may contribute to impaired health status in non-human primates.

## Data Availability

No datasets were generated or analysed during the current study.
